# Terrell Medical Society (Regular Meeting, February 4th, Ult.)

**Published:** 1896-03

**Authors:** James Orr

**Affiliations:** Secretary


James Orr, M. D.,

Committee.

---

### Society Notes.

### Terrell Medical Society (Regular Meeting, February 4th, ult.).

The regular monthly meeting of Terrell Medical Society was held on the 4th ult. Dr. W. H. Monday, the President, in the chair. After the usual business part of the meeting had been transacted, Dr. F. S. White, suggesting the near approach of the

State Association meeting, and advisability of the profession discussing and agreeing upon such legislation as they may deem necessary to aid their great work for humanity, moved to dispense with the regular order of business that such discussion might be had. The motion prevailing, the doctor then offered a resolution setting forth the fact that hundreds of epileptics and chronic insane persons are now in the State without adequate care or protection, many being confined in filthy and often overcrowded jails; demanding that the State make ample preparation for the care of these unfortunate people, and raising a committee to bring the matter to the attention of the medical profession and the people at large. In speaking to his resolution, Dr. White forcibly illustrated the pitiable condition of these poor unfortunates, who should receive all the attention which the dictates of humanity suggest. He advocated the cottage plan of caring for these people, saying it could be established comparatively cheaply, and be made largely self-sustaining through the labors of the occupants.

Dr. Stovall moved to amend the resolution by adding the words ''and inebriates'' after chronic insane, which the author accepted. Dr. Stovall said the time had arrived when all advanced medical men recognized habitual inebriety as a disease, and it was time for them to speak out on the subject, contending that many unfortunate drinkers might thus be saved.

Dr. Orr said he was in favor of both, resolution and amendment, provided the latter was construed to mean inebriates from opium, cocaine, chloral and chloroform, as he thought them as much objects of pity and care as any other form of mentally-defective people. This meeting the approbation of the authors, the doctor said he was an advocate of the adoption of the resolution as amended, for he thought it more important, more humane, to care for these people than to expend State moneys in any other way. He deprecated the fact that all plans for State economy, made by political office seekers, had asylum appropriations for their first point of attack, and the greatest reductions were always made or recommended from their allowance, while courts and pie-eating boards were never stinted. He said he thought the time had arrived when the interests of the tax-payers would be subserved, and the condition of the unfortunate benefited by removing the penal and charitable institutions of the State from the domain of practical politics, that they might no longer be considered the stock in trade of incoming administrations, with which friends were to be rewarded, campaign obligations paid, or refractory opponents punished. To this end he favored the selection of a State board of charities and corrections, not more than two of whom should belong to one political party, to have charge of all our penal and charitable institutions, including the duties now discharged by financial agents of the penitentiaries and the Pardon Board; and he would favor a constitutional amendment making the terms of office of this board six years, so as to remove it from political influence, if possible. This board could do the work now performed by over half a hundred political appointees, and would do it much more economically and with better results than at present.

Again, he favored the appointment of a State Board of Health, one of whom should be a homeopath, whose duty it should be:

First. To transact all business now pertaining to the Health Officer.

Second. To perform the duties now neglected by the fifty odd Medical Examing Boards of the State, examining, under more rigid rules than now exist, every person seeking to practice medicine, whether he be a graduate or not, for which purpose they should hold meetings at different times and places throughout the State.

Third. To have such sanitary control of all public buildings and grounds as may be necessary, and to do the general duties of sanitary inspectors, particular attention being paid to the proper sanitation of public buildings to be hereafter erected, and to necessary hygiene arrangements for existing State institutions, especially its prisons, many of which are an abomination.

Dr. Dumas endorsing the resolution and the last speaker, said it was always hard to get politicians to recognize the right of physicians to be heard even, let alone direct, in matters in which they should be better qualified than any others to suggest, and where professional pride would always cause them to study well before advocating any system or change. The average law maker of to-day resents any suggestions from physicians on medical subjects, and grudgingiy votes appropriations to the poor and unfortunate who are not influential voters. He said we must show the people the economy, advantage and humanity of these measures advocated.

Dr. Anthony, endorsing the resolution and amendments, said he was afraid we would find trouble in getting chronic alcoholic inebriety classed as a disease by legislators, who are so often free users of the poison themselves. He did not wish to ask too much of the legislature and get nothing; however, he recognized the duty of the State to care for mental incapables from whatever cause produced. He said an asylum for the victims of drug habits should be established, in which habitual inebriates could be confined by law and treated free of charge, and to which all victims of drug habits could be admitted and treated at comparatively nominal figures, much less indeed than is now charged by specialists and often unscruplous impostors.

The president taking the floor earnestly advocated the resolution as amended, also the additional legislation advocated by Dr. Orr. He paid a tribute to the profession on account of their charitable and philanthropic work, and the unselfish manner in which all true physicians, practicing medicine as a science, discharge their duties. He wanted this matter brought before the profession of the State first, and then to the attention of all Christian and philanthropic people. Closing he urged medical organization, and recommended all members of this Society who have not already done so, to join the State Medical Association, which meets in Fort Worth in April.

The resolution as amended was then passed unanimously. [Published elsewhere in this issue.—Ed.]

The president appointed Drs. F. S. White, James Orr and C. O. Mathews as the committee.

James Orr, M. D., Secretary.

